# Viability of a MSQOL-54 general health-related quality of life score using bifactor model

**DOI:** 10.1186/s12955-021-01857-y

**Published:** 2021-09-25

**Authors:** Andrea Giordano, Silvia Testa, Marta Bassi, Sabina Cilia, Antonio Bertolotto, Maria Esmeralda Quartuccio, Erika Pietrolongo, Monica Falautano, Monica Grobberio, Claudia Niccolai, Beatrice Allegri, Rosa Gemma Viterbo, Paolo Confalonieri, Ambra Mara Giovannetti, Eleonora Cocco, Maria Grazia Grasso, Alessandra Lugaresi, Elisa Ferriani, Ugo Nocentini, Mauro Zaffaroni, Alysha De Livera, George Jelinek, Alessandra Solari, Rosalba Rosato

**Affiliations:** 1 Unit of Neuroepidemiology, Fondazione IRRCS Istituto Neurologico Carlo Besta, Milan, Italy; 2 Department of Psychology, University of Turin, Turin, Italy; 3 Department of Human and Social Sciences, University of Aosta Valley, Aosta, Italy; 4 Department of Biomedical and Clinical Sciences L. Sacco, Università di Milano, Milan, Italy; 5 Distretto Sanitario di Catania, ASP di Catania, Catania, Italy; 6 Neurology Unit & Regional Referral Multiple Sclerosis Centre (CReSM), University Hospital San Luigi Gonzaga, Orbassano, Italy; 7 Department of Neuroscience, San Camillo-Forlanini Hospital, Rome, Italy; 8 Department of Neurosciences, Imaging and Clinical Sciences, University G. d’Annunzio, Chieti, Italy; 9 Servizio di Psicologia e Neuropsicologia, UO di Neurologia e Riabilitazione Specialistica Neurologica, San Raffaele Hospital, Milan, Italy; 10 Laboratory of Clinical Neuropsychology, Psychology Unit, ASST Lariana, Como, Italy; 11 IRCCS Don Gnocchi Foundation, Florence, Italy; 12 Multiple Sclerosis Center, Neurology Unit, Hospital of Vaio, Fidenza, Italy; 13 Department of Basic Medical Sciences, Neurosciences and Sense Organs, University of Bari, Bari, Italy; 14 Multiple Sclerosis Center, Unit of Neuroimmunology and Neuromuscular Diseases, Fondazione IRRCS Istituto Neurologico Carlo Besta, Milan, Italy; 15 Department of Medical Science and Public Health, University of Cagliari, Cagliari, Italy; 16 Multiple Sclerosis Center, ASSL Cagliari, ATS Sardegna, Cagliari, Italy; 17 Multiple Sclerosis Unit, IRCCS S. Lucia Foundation, Rome, Italy; 18 IRCCS Istituto delle Scienze Neurologiche di Bologna, Bologna, Italy; 19 Dipartimento di Scienze Biomediche e Neuromotorie, Università di Bologna, Bologna, Italy; 20 UOC Psicologia Ospedaliera, AUSL di Bologna, Bologna, Italy; 21 Department of Clinical Sciences and Translational Medicine, University of Rome “Tor Vergata”, Rome, Italy; 22 Neurorehabilitation Unit 3, IRCCS S. Lucia Foundation, Rome, Italy; 23 Multiple Sclerosis Centre, ASST Valle Olona, Gallarate, Italy; 24 Neuroepidemiology Unit, Centre for Epidemiology and Biostatistics, Melbourne School of Population and Global Health, The University of Melbourne, Melbourne, Australia; 25 Unit of Clinical Epidemiology, “Città della Salute e della Scienza” Hospital and CPO Piemonte, Turin, Italy

**Keywords:** Multiple sclerosis, Bifactor model, Dimensionality, Factor analyses, Health-related quality of life, MSQOL-54

## Abstract

**Background:**

MSQOL-54 is a multidimensional, widely-used, health-related quality of life (HRQOL) instrument specific for multiple sclerosis (MS). Findings from the validation study suggested that the two MSQOL-54 composite scores are correlated. Given this correlation, it could be assumed that a unique total score of HRQOL may be calculated, with the advantage to provide key stakeholders with a single overall HRQOL score. We aimed to assess how well the bifactor model could account for the MSQOL-54 structure, in order to verify whether a total HRQOL score can be calculated.

**Methods:**

A large international database (3669 MS patients) was used. By means of confirmatory factor analysis, we estimated a bifactor model in which every item loads onto both a general factor and a group factor. Fit of the bifactor model was compared to that of single and two second-order factor models by means of Akaike information and Bayesian information criteria reduction. Reliability of the total and subscale scores was evaluated with Mc Donald’s coefficients (omega, and omega hierarchical).

**Results:**

The bifactor model outperformed the two second-order factor models in all the statistics. All items loaded satisfactorily (≥ 0.40) on the general HRQOL factor, except the sexual function items. Omega coefficients for total score were very satisfactory (0.98 and 0.87). Omega hierarchical for subscales ranged between 0.22 to 0.57, except for the sexual function (0.70).

**Conclusions:**

The bifactor model is particularly useful when it is intended to acknowledge multidimensionality and at the same time take account of a single general construct, as the HRQOL related to MS. The total raw score can be used as an estimate of the general HRQOL latent score.

**Supplementary Information:**

The online version contains supplementary material available at 10.1186/s12955-021-01857-y.

## Introduction

Over the last two decades, health related quality of life (HRQOL) measures have been increasingly included into research studies of neurodegenerative disorders, including multiple sclerosis (MS) [1–3]. Importantly, HRQOL instruments can disclose aspects of disease which are not considered by standard clinical tools, and that would otherwise go unrecognized. In addition, HRQOL instruments can help clinicians appreciate patient priorities particularly in terms of treatment goals, facilitate physician–patient communication, and promote shared decision making [4].

The Multiple Sclerosis Quality of Life-54 items (MSQOL-54) inventory was designed to address the need for HRQOL measures to be used in quality of care and clinical effectiveness research. Thus, the MSQOL-54 comprehensively assesses the HRQOL of patients with MS, an unpredictable chronic neurological disorder which affects 2.8 million people worldwide [5, 6].

Compared to other instruments, its main strength is that it combines a generic- and a disease-targeted approach. In fact, the MSQOL-54 is a multidimensional, MS-specific HRQOL instrument, based on the generic SF-36 [7] supplemented with 18 MS-specific items [8]. This approach allows to compare HRQOL in MS with that in other diseases and with the general population using the generic score, in addition to allowing a sensitive measure for within-disease comparisons.

It consists of 52 items combined in 12 subscales, and two single items. Two composite scores (Mental Health Composite, MHC, and Physical Health Composite, PHC) are determined by aggregating scores of the pertinent subscales [8]. Psychometric properties like construct and content reliability, discrimination [9–11], and responsiveness [12] have been rigorously documented. It was developed in US English, and clinically validated in various languages [9–11, 13–16], including Italian [9]. Despite these key advantages, MS patients were not involved in its development [8].

In the validation work of the MSQOL-54, Vickrey et al. [8] reported a quite high correlation (r = 0.66) between the two composite scores. Given this correlation, it could be hypothesized that a unique total score of HRQOL may be calculated, with the benefit to provide patients, clinicians and researchers with a single overall HRQOL assessment, to assess for example, treatment response or modify treatment plan. In this very context, applying a bifactor model to the MSQOL-54 items could be particularly useful, as it is intended to acknowledge multidimensionality and, at the same time, take account of a single general construct [17], as the HRQOL related to MS is. The bifactor model may constitute an alternative to the more widely-used second-order models, or correlated-traits [18]. By definition, the bifactor model is employed so that each item loads on a general factor and only one group factor, and the general and group factors are all uncorrelated to each other [18]. For each single item, the general factor captures what the item shares with all the other items and the group factor reflects what the item shares with the other items belonging to the same subscale, once the influence of the general factor has been removed. That is, all the covariation between items and all the covariation between subscale scores is captured by the general factor that is a broad latent dimension made of all the subscale contents. Bifactor modeling is generally used to test multifaceted constructs [17], and so far, has been used mainly in the area of intelligence research [19, 20], and in the study of personality [21, 22]. However, this has rarely been applied in neurology and MS research, except for a few studies [23–25].

In the present study, our primary aim was to apply the bifactor model to the MSQOL-54 items in order to verify whether a total HRQOL score could be calculated. Second, if the bifactor model fitted the data well, we aimed to evaluate the measurement invariance of MSQOL-54 items across age and gender.

## Methods

### Participants

To perform the present secondary analysis, we used data drawn from different datasets collected utilizing the MSQOL-54 within ongoing or completed projects conducted in Italy and Australia [26].

We obtained the data collected with the English version from the *‘HOLISM study’*, an observational international study, whose methods and results have been reported elsewhere [27, 28]. Briefly, participants from Europe, Australasia, North America, and other countries were recruited in 2014 via online platforms (e.g. websites, and forums involving MS patients, and social media). The study aimed to provide an overview of risk-modifying behaviors and current lifestyle of a large international cohort of MS patients to analyze the association between these variables and disease progression. Patients with ≥ 18 years, and who could undertake an English language survey were included. In the present study, we used baseline data from English-speaking countries only: 840 (41%) from North America, 797 (39%) from Australasia, and 427 (20%) from UK and Ireland.

We obtained the data collected with the Italian version from the datasets (i.e. baseline data for longitudinal studies/trials) of the following research projects:

- The *‘Care system project’* [29, 30], an observational study aimed to assess MS patients’ perceived levels of well-being and ill-being (overall, 662 MS patients from 8 MS centers, recruited between 2012 and 2017). Patients with ≥ 18 years, having a clinically-definite MS diagnosis [31] for at least 3 years, and having a caregiver, were included. Patients with neurological disorders other than MS, psychiatric disorders, Expanded Disability Status Scale (EDSS [32]) ≥ 8, being in the active phase of MS, severe cognitive impairment, were excluded.
- The study *‘An abbreviated computerized version of the MSQOL-54: Development and preliminary validation using Confirmatory Factor Analysis and Item Response Theory’* [33, 34], which developed an abbreviated version of the MSQOL-54. We used data from 564 MS patients recruited at 5 MS centers, between 2005 and 2012 who participated in the retrospective phase of the study [33]. Patients with ≥ 18 years, having a clinically-definite MS diagnosis and fluent in Italian, were included.
- Other research projects carried out in 5 Italian MS centers. Patients with ≥ 18 years, having a clinically-definite MS diagnosis able to read and understand Italian, were included. Overall, 379 MS patients, recruited between 2005 and 2017, were included in the present study.

All these projects were approved by local ethics committees (St Vincent’s Hospital Melbourne Human Research Ethics Committee [LRR 055/12]; Università di Milano; San Raffaele Hospital, Milano; University Polyclinic Hospital G. Rodolico, Catania; University of Florence; S. Anna Hospital, Como; Hospital of Vaio-Fidenza, Fidenza; University ‘G. D’Annunzio’, Chieti; University of Bari; San Camillo- Forlanini Hospital, Rome; University Hospital ‘San Luigi Gonzaga’, Orbassano; Fondazione IRCCS Istituto Neurologico ‘C. Besta’, Milano; IRCCS S. Lucia Foundation, Rome). Patients gave written or online informed consent to be included in the original projects. Additional consent was not required for this secondary analysis, for which patients’ privacy and anonymity were guaranteed.

Records were included in the database if the following variables were available: MS diagnosed (according to any criteria, Italian sample) or disclosed by a physician (English-speaking sample); patient age ≥ 18 years; gender; level of disability (EDSS, Italian sample; PDDS [35], English-speaking sample), and disease duration.

### Statistical analysis

The goodness of fit of the original second-order factor model comprising two factors, the novel second-order factor model comprising one factor, and the bifactor model was tested using confirmatory factor analysis (CFA).

According to the original factor structure of the MSQOL-54, in the two second-order factor model, it was hypothesized that 52 items loaded in 12 first-order factors and two second-order factors, corresponding to the PHC and MHC [8] (Additional file 1). The remaining two items (i.e. item 2 *‘Compared to one year ago, how would you rate your health in general now?’,* and item 50 *‘Overall, how satisfied were you with your sexual function during the past 4 weeks?’*) were not included in this model, as well in the other models, because they are single items.

In the single second-order factor model, the first-order factors were the same as in the original model, and one second-order factor was imposed, called ‘HRQOL’ (Additional file 2).

In the bifactor model, it was hypothesized that 50 items loaded onto the general HRQOL factor and on their specific group factors, whereas the two items forming the overall QOL subscale (items 53 and 54) were loaded only onto the general factor, because the bifactor model needs each group factor to be composed of at least three items to be identified (Additional file 3).

Global fit of the models was evaluated with three approximate indices recommended by Kline [36], namely, the root mean square error of approximation (RMSEA), the comparative fit index (CFI), and the standardized root mean square residual (SRMR). As a rule of thumb, RMSEA under 0.08 represents good fit and values below 0.05 represent very good fit [37]; SRMR values under 0.08 indicate good fit, and values greater than 0.10 indicate poor fit [36]; concerning CFI, values above 0.95 are indicative of good fit [38], and, as for other incremental fit indices, values below 0.90 indicate that models “can usually be improved substantially” [39]. Akaike Information Criterion (AIC) [40] and Bayesian Information Criterion (BIC) [41, 42] were used for model comparisons. The model with lower AIC and BIC values was chosen as the best model to fit the data.

To evaluate the relative strength of the general HRQOL factor to group factors, magnitude of loadings was considered (values ≥ 0.40 were considered satisfactory [43], and explained common variance (ECV) and percentage of uncontaminated correlations (PUC) were calculated [44]. A high ECV value or a moderate ECV value supplemented with a high PUC value (> 0.90) indicated that data were sufficiently “unidimensional” [45]. To judge the degree to which total raw scores reflected a common single factor, the McDonald’s coefficient omega hierarchical (ω_H_) was computed. High values meant that the total raw score was a reliable measure of the general factor. Further, to evaluate the reliability considering all sources of common variance (general and group factor), the McDonald’s coefficient omega (ω) was calculated. Both omega hierarchical and omega were also calculated for each subscale to evaluate how much subscale scores were reliable measures of the corresponding specific latent variables, once items’ common variance due to the general factor was removed (ω_S_), and how reliable they were considering all sources of common variance.

Finally, we used CFA to evaluate the measurement invariance of MSQOL-54 across gender (male [26%]; female [74%]), and age (using the median of 44 years old as cut-off). Three increasingly constrained levels of measurement invariance (i.e. configural, metric, scalar) were assessed using multi-group CFA. We used the same criteria as above to assess the model fit.

In line with Chen [45], a worsening of CFI exceeding the cut-off of 0.010, accompanied by a change of ≥ 0.030 in SRMR or a change of ≥ 0.015 in RMSEA was deemed a signal of lack of metric invariance; as regards the scalar invariance, the threshold values for RMSEA and CFI were identical to those used for metric invariance, whereas it was 0.010 for SRMR. To liken the fit of two nested models, the χ2 difference test was not employed, as it is responsive to sample size, therefore usually providing significant results with large sample sizes [45].

All models were estimated using the software Mplus 7.0 with the maximum likelihood estimation with robust standard errors (MLR) [46].

## Results

The database consisted of 3669 MS patients (mean age 43.8 years [range 18–87], 74% women, 54% with a mild level of disability (measured with the self-reported PDDS), and mean disease duration of 7.2 years [0–48]) (Table 1). Of these, 1605 (44%) were Italian (mean age 40.9 years, 62% women, 69% with a mild disability level) and 2064 English-speaking participants (mean age 46.1 years, 83% women, 54% with a mild disability level). Compared to Italians, English-speaking participants were older, had a higher percentage of women, and had longer disease duration (*p* < 0.001) (Table 1).

**Table 1:** Characteristics of the entire dataset (N = 3669 patients) and of the English-speaking and Italian samples

	English-speaking (N = 2064)	Italian (N = 1605)	Total sample (N)
Women (%)	1704 (83)	996 (62)	2700 (74)
Mean age in years, SD (range)	46.1, 10.5 (18–87)	40.9, 10.8 (18–79)	43.8, 10.9 (18–87)
Mean years from MS diagnosis, SD (range)	9.0, 7.3 (1–42)	4.9, 7.8 (0–48)	7.2, 7.8 (0–48)
Median EDSS score (range)	–	2.5 (0–9.5)	2.5 (0–9.5)
*PDDS (%)*			
Mild disability	1110 (54)	1097 (69)	1110 (54)
Moderate disability	722 (35)	308 (19)	722 (35)
Severe disability	219 (11)	194 (12)	219 (11)
Mean MSQOL-54 PHC, SD (range)	57.7, 21.5 (3–100)	61.1, 20.2 (2–100)	59.2, 21.1 (1–100)
Mean MSQOL-54 MHC, SD (range)	66.6, 21.3 (1–100)	62.9, 20.7 (2–100)	65.0, 21.1 (1–100)

EDSS, Expanded Disability Status Scale; MSQOL-54, Multiple Sclerosis Quality of Life-54; PDDS, Patient Determined Disease Steps; PHC/MHC, Physical and Mental Health Composite; SD standard deviation

The goodness-of-fit statistics of the three alternative CFA models are reported in Table 2.

**Table 2:** Model description and fit statistics of confirmatory factor analysis

Model type	DF	AIC	BIC	RMSEA	CFI	SRMR
Two second-order factors ^a^	1260	1,711,214	1,712,270	0.055	0.888	0.064
Single second-order factor ^b^	1262	1,711,735	1,712,778	0.056	0.884	0.068
Bifactor 1^c^	1223	[1710635]	[1711920]	[0.055]	[0.892]	[0.062]
Bifactor 2^d^	1225	1,710,637	1,711,910	0.055	0.892	0.062

AIC, Akaike information criterion; BIC, Bayesian information criterion; RMSEA, root mean square error of approximation; CFI, comparative fit index; DF, Error degree of freedom; SRMR, standardized root mean square residual

^a^ 12 first-order factors and two second-order factors; the correlation between the two second-order factors was 0.87

^b^ 12 first-order factors and one second-order factor

^c^ 11 group factors and one general factor; residual of overall quality of life (QOL) subscale items (items 53 and 54) were allowed to correlate. Solution not admissible as item 20 showed a negative variance

^d^ 10 group factors and one general factor; items of the social function subscale (20, 33, 51) loaded onto the general factor only, and residual of the overall QOL subscale items (53, 54), and the residual of items 20 and 33 of the social function subscale were allowed to correlate

The (original) two second-order factor model fitted the data quite well (RMSEA = 0.055; CFI = 0.888, SRMR = 0.064), only the CFI index was slightly under the cut-off value. The single second-order factor model showed similar values (RMSEA = 0.056; CFI = 0.884, RMRS = 0.068), but in terms of AIC and BIC values it was outperformed by the two second-order factor model. The bifactor model (‘bifactor 1’) produced apparently good fit measures, but the solution was inadmissible because one item of the social function subscale (item 20 ‘*During the past 4 weeks, to what extent has your physical health or emotional problems interfered with your normal social activities with family, friends, neighbors, or groups’*) showed a negative residual variance (Additional file 4). An inspection of the loading estimates revealed that one item (item 51 *‘During the past 4 weeks, to what extent have problems with your bowel or bladder function interfered with your normal social activities with family, friends, neighbors, or groups?’*) was not a good indicator of social functioning, once parceling out the general factor. In fact, a supplementary analysis conducted with the three items of the social function subscale showed that the zero order correlations between item 51 and items 20 and 33 were 0.40 and 0.39, respectively. Further, partial correlations between the same items, after controlling for HRQOL subscale score, were lower (0.24 and 0.22, respectively) (Additional file 5**).** Therefore, the bifactor 1 solution was inadmissible, being necessary to re-specify a second bifactor model. In the ‘bifactor 2’ the three items of the social function subscale (20, 33, and 51) loaded onto the general factor only, and, to account for the group specificity of item 20 and item 33, residuals of these two items were allowed to correlate. This last model had satisfactory fit (RMSEA = 0.055; CFI = 0.892, RMRS = 0.062), and both AIC and BIC statistic values were better than those of the one and two second-order factor models (AIC = 1,710,637; BIC = 1,711,910; Table 2).

Standardized factor loadings for the revised bifactor model are shown in Table 3.

**Table 3:** Standardized factor loadings in the bifactor model (Bifactor 2)

Scales	Items	Factor loading	
		General HRQOL factor	Group factor
Physical function	3. Vigorous activities	0.553	0.445
	4. Moderate activities	0.594	0.622
	5. Lift, carry groceries	0.554	0.620
	6. Climb several flights	0.569	0.665
	7. Climb one flight	0.533	0.695
	8. Bend, kneel	0.551	0.594
	9. Walk mile	0.555	0.669
	10. Walk several blocks	0.526	0.734
	11. Walk one block	0.488	0.726
	12. Bath, Dress	0.461	0.523
Role limitations due to physical problems	13. Cut down time	0.542	0.537
	14. Accomplished less	0.571	0.571
	15. Limited in kind	0.581	0.645
	16. Had difficulty	0.594	0.586
Role limitations due to emotional problems	17. Cut down time	0.504	0.646
	18. Accomplished less	0.515	0.699
	19. Not careful	0.509	0.592
Bodily pain	21. Pain magnitude	0.575	0.702
	22. Pain interfere with work	0.611	0.653
	52. Pain interfere with enjoyment	0.601	0.652
Emotional wellbeing	24. Nervous person	**0.371**	0.531
	25. Down in dumps	0.561	0.585
	26. Peaceful	0.562	**0.369**
	28. Blue/Sad	0.594	0.592
	30. Happy	0.535	0.432
Energy	23. Pep/life	0.713	**0.206**
	27. Energy	0.717	**0.245**
	29. Worn out	0.624	0.546
	31. Tired	0.620	0.602
	32. Rested on walking in the morning	0.519	**0.281**
Health perceptions	1. EVGFP rating	0.638	0.452
	34. Sick easier	0.417	**0.269**
	35. As healthy	0.463	0.575
	36. Health to get worse	0.450	**0.233**
	37. Health excellent	0.590	0.659
Cognitive function	42. Concentration and thinking	0.591	0.710
	43. Sustained attention	0.576	0.700
	44. Memory	0.467	0.708
	45. Others note troubles with memory/concentration	0.436	0.564
Health distress	38. Discouraged	0.729	0.508
	39. Frustrated	0.712	0.544
	40. Worried for life	0.624	0.543
	41. Weighed down	0.694	0.563
Sexual function	46. Lack if sexual interest	**0.346**	0.684
	47. Erection/Lubrication	**0.299**	0.758
	48. Orgasm	**0.348**	0.724
	49. Satisfy sexual partner	**0.378**	0.656
Social function	20. Social extent, physical health	0.737	–
	33. Social time	0.758	–
	51. Social extent, bowel or bladder	0.505	–
Overall quality of life	53. 0–10 NRS rating	0.735	–
	54. TUMMMPO rating	0.685	–

EVGFP, Excellent, Very good, Good, Fair, Poor. HRQOL, health-related quality of life. NRS, Numeric Rating Scale. TUMMMPO, Terrible, Unhappy, Mostly dissatisfied, Mixed—about equally satisfied and dissatisfied, Pleased, Delighted

Correlations between residuals: 0.524 (items 53 and 54); 0.411 (items 20 and 33)

Coefficients < 0.40 are reported in bold; all the loadings are statistically significant at *p* < 0.001

All items loaded satisfactorily on the general (HRQOL) factor (loading ≥ 0.40), the only exception being item 24 (*‘Have you been a very nervous person?’*), and the four items belonging to the sexual function scale.

Loadings on the group factors were all ≥ 0.40, except for three items (item 23 *‘Did you feel full of pep?’*, item 27 *‘Did you have a lot of energy?’*, and item 32 *‘Did you feel rested on waking in the morning?’*) of the energy subscale, two items of health perceptions (i.e. item 34 *‘I seem to get sick a little easier than other people’* and item 36 *‘I expect my health to get worse’*), and one of the emotional wellbeing subscale (item 26 *‘Have you felt calm and peaceful’*).

ECV value was 0.51 (indicating that 51% of the common variance was due to the general HRQOL factor) and PUC was 0.92, denoting that the data were sufficiently ‘unidimensional’.

Omega value for the total raw score was 0.98, suggesting that the reliability considering all sources of common variance (general factor and group factors) was very high. Moreover, omega hierarchical value of the general factor was 0.87, indicating that the total raw score was a reliability measure of the general HRQOL factor.

As shown in Table 4, for the majority of the subscales, omega hierarchical value (ω_S_) was around 0.50, whereas it was very low (≤ 0.35) for three subscales (i.e. energy, health perceptions, and health distress)—meaning that summed scores of items belonging to these subscales were not a reliable measure of their respective domain latent variable once the general HRQOL was taken into account—and it was high (0.70) for sexual function subscale. For the latter subscale, it seems that the specific group factor accounted for more variance than the general factor, indicating that items belonging to this subscale were more likely to reflect a specific domain of HRQOL (related to sexual function) than a common general construct of HRQOL.

**Table 4:** Omega statistics for the MSQOL-54 total and subscales scores

Subscale	No. of items	ω	ω_S_
Physical function	10	0.96	0.55
Role limitations due to physical problems	4	0.89	0.46
Role limitations due to emotional problems	3	0.86	0.53
Bodily pain	3	0.92	0.52
Emotional wellbeing	5	0.85	0.41
Energy	5	0.86	0.22
Health perceptions	5	0.82	0.35
Cognitive function	4	0.91	0.57
Health distress	4	0.93	0.35
Sexual function	4	0.87	0.70

ω = scores reliability considering all sources of common variance (the general and the group factor); ω_S_ (omega hierarchical subscale) = scores reliability considering only the common variance due to the group factor, that is the reliability of subscales scores, controlling for the effects of the general factor

### Measurement invariance

First, the model was estimated to evaluate the measurement invariance of MSQOL-54 across gender (Table 5, upper part). Results showed that the model produced an acceptable fit for configural invariance (RMSEA = 0.055; CFI = 0.892; SRMR = 0.063). Considering the model where loadings were imposed to be identical across gender, indices of fit were satisfactory, and worsening of the unrestrained model was insignificant (ΔRMSEA < 0.001; ΔCFI =  − 0.006; ΔSRMR = 0.008), hence providing evidence of metric invariance. With regard to the scalar invariance (i.e. intercepts and loadings imposed to be invariant across groups), the model fitted the data well (RMSEA = 0.054; CFI = 0.885; SRMR = 0.063). Finally, examining the variations in fit indices when compared with the metric invariance model, cut-off values were met, supporting the scalar invariance.

**Table 5:** Measurement invariance of MSQOL-54 across gender and age

	χ^2^ (df)^a^	RMSEA	CFI	SRMR	ΔRMSEA	ΔCFI	ΔSRMR
Male	4658.7 (1225)	0.054	0.891	0.063			
Female	11,160.1 (1225)	0.055	0.893	0.062			
Configural invariance	15,829.7 (2450)	0.055	0.892	0.063			
Metric invariance	16,126.6 (2538)	0.054	0.891	0.065	− 0.001	− 0.001	0.002
Scalar invariance	16,598.8 (2579)	0.055	0.887	0.065	0.001	− 0.004	0.000
Adults < 44 years old	7253.1 (1225)	0.053	0.890	0.056			
Adults ≥ 44 years old	7811.9 (1225)	0.054	0.895	0.061			
Configural invariance	15,047.9 (2450)	0.054	0.893	0.059			
Metric invariance	15,588.1 (2538)	0.054	0.889	0.063	0.000	− 0.004	0.004
Scalar invariance	16,084.7 (2579)	0.054	0.885	0.063	0.000	− 0.002	− 0.004

CFI, comparative fit index; df, degrees of freedom; RMSEA, root mean square error of approximation; SRMR, standardized root mean square residual

^a^ χ^2^ *p*-values are all < 0.001

Second, the model was estimated to evaluate the measurement invariance of MSQOL-54 across age (using the median of 44 years as cut-off) (Table 5, bottom part). Here, the results showed that the model produced acceptable fit for configural invariance (RMSEA = 0.054; CFI = 0.893; SRMR = 0.059), metric invariance (RMSEA = 0.054; CFI = 0.887; SRMR = 0.067), and scalar invariance (RMSEA = 0.054; CFI = 0.885; SRMR = 0.063). All the changes in fit indices across the models were satisfactory.

## Discussion

As far as we know, this was the first study applying the bifactor model to the MSQOL-54 in a large international database of MS patients.

The bifactor model with one general HRQOL factor and 10 specific group factors achieved acceptable fit and outperformed both the original two second-order factor model and the single second-order factor model. Also, our findings supported measurement invariance of the questionnaire across age and gender, suggesting that it has the same meaning across these socio-demographic variables, and that patients having the same ratings on MSQOL-54 general or domain factors would attain the identical value on the observed variable, regardless of sub-group membership.

Generally, the factor loadings were substantially high both on the general and the group factors, and the ECV was about 50%, indicating that MSQOL-54 items contribute to essentially the same extent to both the general HRQOL factor and to the group factors. Despite this, the data can be deemed sufficiently ‘unidimensional’, because the MSQOL-54 consists of several subscales composed of few items each, and this implies that the vast majority of correlations between items (PUC = 92%) reflect general factor variance only. Furthermore, the satisfactory value of the coefficient omega hierarchical indicated that the total raw score is a reliable measure of the general HRQOL latent variable. Taken together, all these results support the hypothesis that the MSQOL-54 has a sufficient ‘unidimensional’ structure, and thus it is appropriate to calculate a total HRQOL score.

Among the 52 items analyzed in the study—it is noteworthy to remember that items 2 and 50 were excluded from the analysis as they are single items—the weaker indicators of the general HRQOL dimension were the four items of the sexual function subscale. Considering the omega hierarchical value, the sexual function subscale is more likely to reflect a specific domain of HRQOL (namely related to sexual function) than a common general construct of HRQOL. In fact, this is the only subscale that showed an omega hierarchical value ≥ 0.70.

Another issue derives from the social function subscale. The three items of this subscale loaded onto the general factor only because one of them (item 51, dealing with bowel or bladder) was not a good indicator of social functioning, and a group factor needs at least 3 items to be identified. Thus, it was not possible to evaluate the contribution of the relative group factor.

This study has important implications for clinical practice and research. For clinical practice, it could be crucial to provide health professionals and MS patients with feedback using a single HRQOL total score, which includes aspects of HRQOL not captured by the 10 group factors—as well as with subscale scores, to add granularity. The total HRQOL score could be useful also to identify patient subgroups—with different disease forms as well as levels of disability—in order to deliver personalized interventions addressing, for example, self-efficacy or resilience. On the other hand, for researchers, it could be easier to calculate and interpret a unique total HRQOL score, when using such measure in clinical trials or other research studies. Moreover, the present results can be a stimulus for future research aimed at revising the MSQOL-54 questionnaire. Specifically, our findings highlight the need to enlarge the number of items measuring the social function subscale, because one of the three items of this subscale was not a good indicator. Furthermore, we suggest revising the sexual function subscale items by broadening the content domain so as to include also intimacy and sexual pleasure, as three of the four items from this subscale originated from Medical Outcomes Study sexuality functioning scale which focus on performance indicators [47].

In the present study there were a number of limitations, some of which are reported elsewhere [26]. This secondary analysis was carried out in a large cross-sectional international MS database and should be confirmed in an independent sample, using a prospective longitudinal design. Stability of the factor structure was not established, as the data were not collected using longitudinal assessments. Further, criterion validity of the total HRQOL score should be assessed by correlating it with other pertinent questionnaires.

## Conclusions

To conclude, this study adds new knowledge to the factorial structure of the MSQOL-54, in that a bifactor model fits the data well, outperforming the two second-order models. Therefore, it is appropriate to calculate a total HRQOL score, including all the original subscales/domains. Based on these results, in future research, items should be calibrated using item response theory in order to assess whether a multidimensional computerized adaptative version of the MSQOL-54 is feasible. Further work to integrate / revise selected items is suggested.

## Supplementary Information

**Additional file 1: Supplementary figure 1.** Configuration of the confirmatory two second-order factors model including 12 first-order factors and two second-order factors. CF, cognitive function; EB, emotional wellbeing; EN, energy; HD, health distress; HP, health perceptions; HRQOL, health-related quality of life; PH, physical health; QOL, overall quality of life; RLEP, role limitations due to emotional problems; RLPP, role limitations due to physical problems; SeF, sexual function; SF, social function.

**Additional file 2: Supplementary figure 2.** Configuration of the confirmatory single second-order factor model, including 12 first-order factors and one second-order factor. CF, cognitive function; EB, emotional wellbeing; EN, energy; HD, health distress; HP, health perceptions; HRQOL, health-related quality of life; PH, physical health; QOL, overall quality of life; RLEP, role limitations due to emotional problems; RLPP, role limitations due to physical problems; SeF, sexual function; SF, social function.

**Additional file 3: Supplementary figure 3.** Configuration of the Bifactor 1 model, including 11 group factors and one general factor; items of the overall QOL subscale (53, 54) loaded onto the general factor only, and their residuals were allowed to correlate. CF, cognitive function; EB, emotional wellbeing; EN, energy; HD, health distress; HP, health perceptions; HRQOL, health-related quality of life; PH, physical health; QOL, overall quality of life; RLEP, role limitations due to emotional problems; RLPP, role limitations due to physical problems; SeF, sexual function; SF, social function.

**Additional file 4: Supplementary table 1.**Standardized factor loadings in the Bifactor 1 model.

**Additional file 5: Supplementary table 2.** Correlations between items of the social function subscale.

## Data Availability

The dataset generated and analysed during the current study is available in the Zenodo repository, https://doi.org/10.5281/zenodo.4591136.

## Abbreviations

AIC: Akaike Information Criterion
BIC: Bayesian Information Criterion
CFA: Confirmatory factor analysis
CFI: Comparative fit index
ECV: Explained common variance
EDSS: Expanded Disability Status Scale
HRQOL: Health-related quality of life
MS: Multiple sclerosis
MHC: Mental Health Composite
MLR: Maximum likelihood estimation with robust standard errors
MSQOL-54: The Multiple Sclerosis Quality of Life-54 items
PDDS: Patient Determined Disease Steps
PUC: Percentage of uncontaminated correlations
PHC: Physical Health Composite
RMSEA: Root mean square error of approximation
SRMR: Standardized root mean square residual
US: United States
