# Supplementary figures and images for: Viability of a MSQOL-54 general health-related quality of life score using bifactor model
Source: Health Qual Life Outcomes. 2021 Sep 25;19:224. doi: 10.1186/s12955-021-01857-y (PMC8467164; doi:10.1186/s12955-021-01857-y)

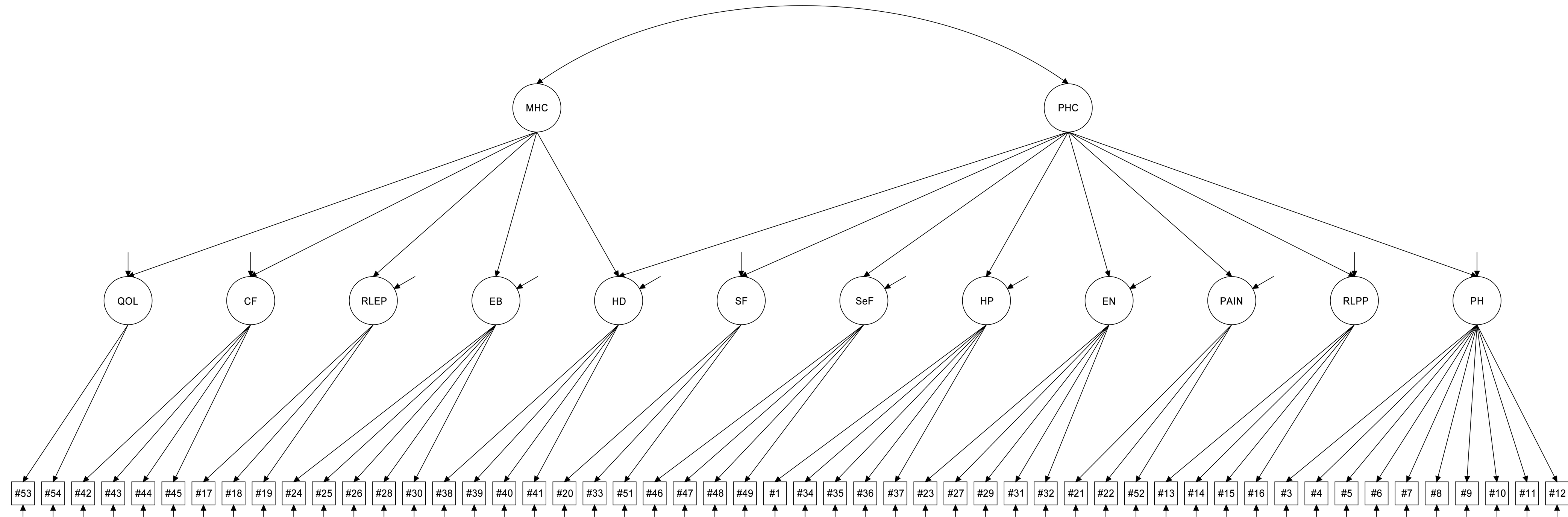

Supplement: Supplementary file 1 — Additional file 1: Supplementary figure 1. Configuration of the confirmatory two second-order factors model including 12 first-order factors and two second-order factors. CF, cognitive function; EB, emotional wellbeing; EN, energy; HD, health distress; HP, health perceptions; HRQOL, health-related quality of life; PH, physical health; QOL, overall quality of life; RLEP, role limitations due to emotional problems; RLPP, role limitations due to physical problems; SeF, sexual function; SF, social function. [file 12955_2021_1857_MOESM1_ESM.pdf]

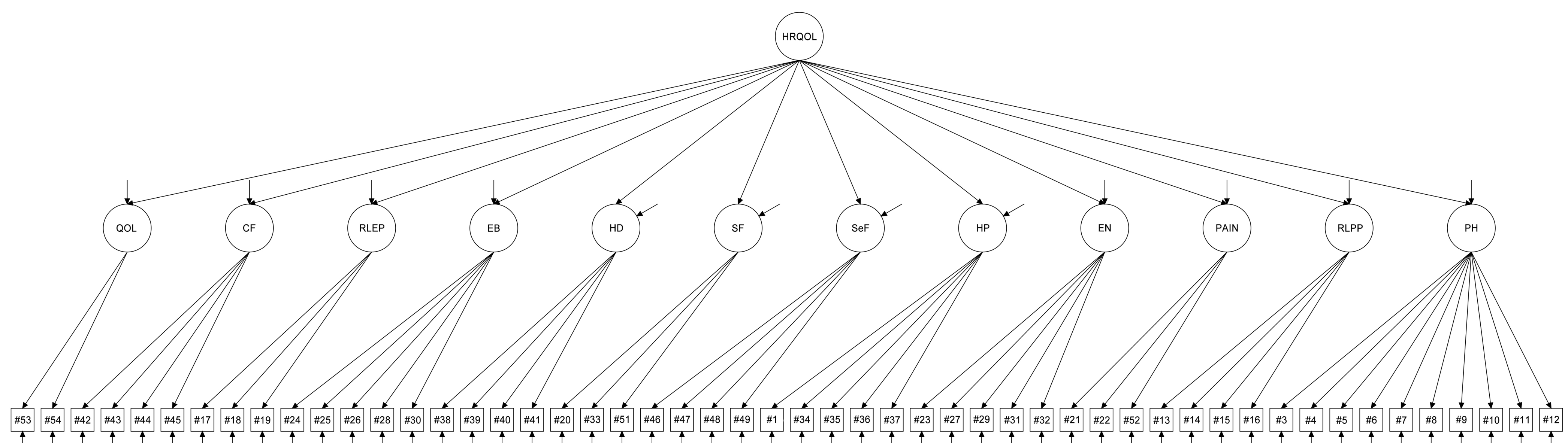

Supplement: Supplementary file 2 — Additional file 2: Supplementary figure 2. Configuration of the confirmatory single second-order factor model, including 12 first-order factors and one second-order factor. CF, cognitive function; EB, emotional wellbeing; EN, energy; HD, health distress; HP, health perceptions; HRQOL, health-related quality of life; PH, physical health; QOL, overall quality of life; RLEP, role limitations due to emotional problems; RLPP, role limitations due to physical problems; SeF, sexual function; SF, social function. [file 12955_2021_1857_MOESM2_ESM.pdf]

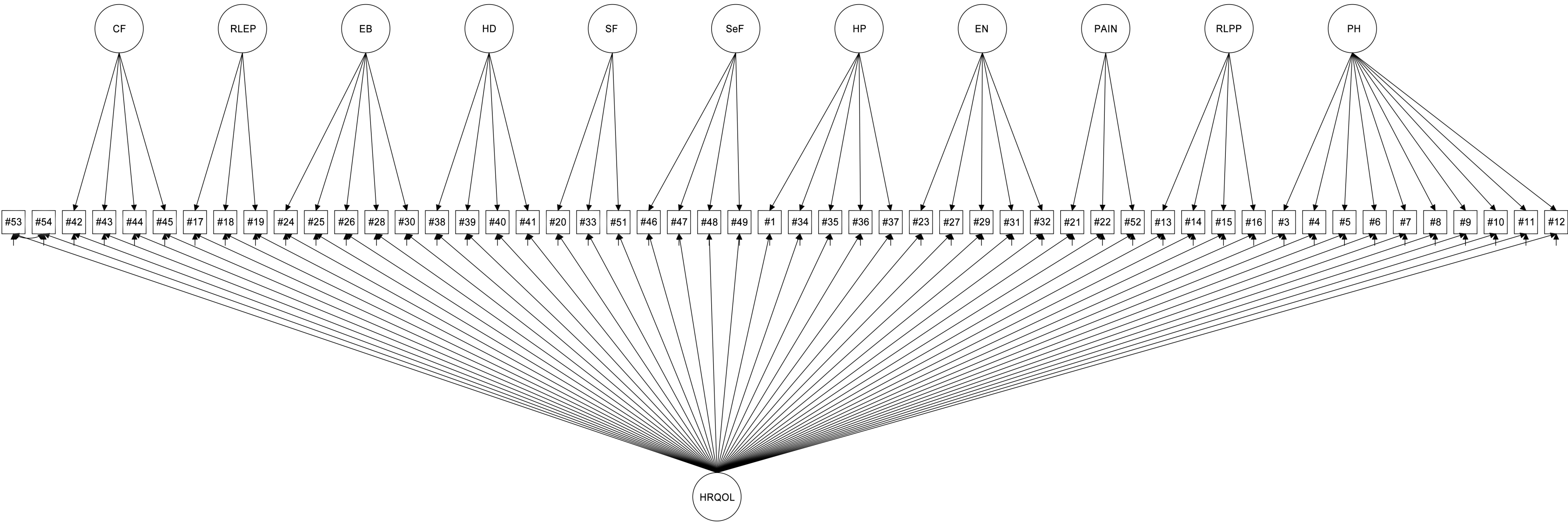

Supplement: Supplementary file 3 — Additional file 3: Supplementary figure 3. Configuration of the Bifactor 1 model, including 11 group factors and one general factor; items of the overall QOL subscale (53, 54) loaded onto the general factor only, and their residuals were allowed to correlate. CF, cognitive function; EB, emotional wellbeing; EN, energy; HD, health distress; HP, health perceptions; HRQOL, health-related quality of life; PH, physical health; QOL, overall quality of life; RLEP, role limitations due to emotional problems; RLPP, role limitations due to physical problems; SeF, sexual function; SF, social function. [file 12955_2021_1857_MOESM3_ESM.pdf]
