# Supplementary material for: Viability of a MSQOL-54 general health-related quality of life score using bifactor model
Source: Health Qual Life Outcomes. 2021 Sep 25;19:224. doi: 10.1186/s12955-021-01857-y (PMC8467164; doi:10.1186/s12955-021-01857-y)
Supplement: Supplementary file 4 — Additional file 4: Supplementary table 1.Standardized factor loadings in the Bifactor 1 model. [file 12955_2021_1857_MOESM4_ESM.pdf]

Additional File 4

Supplementary table 1. Standardized factor loadings in the first bifactor model (Bifactor 1).

Scales	Items	Factor loading	
		General HRQOL factor	Group factor
**Physical function**	3. Vigorous activities	0.552	0.446
	4. Moderate activities	0.593	0.623
	5. Lift, carry groceries	0.553	0.621
	6. Climb several flights	0.568	0.666
	7. Climb one flight	0.532	0.696
	8. Bend, kneel	0.550	0.595
	9. Walk mile	0.554	0.670
	10. Walk several blocks	0.525	0.734
	11. Walk one block	0.487	0.727
	12. Bath, Dress	0.460	0.524
**Role limitations due to physical problems**	13. Cut down time	0.541	0.537
	14. Accomplished less	0.571	0.572
	15. Limited in kind	0.580	0.645
	16. Had difficulty	0.594	0.586
**Role limitations due to emotional problems**	17. Cut down time	0.504	0.646
	18. Accomplished less	0.515	0.699
	19. Not careful	0.509	0.592
**Bodily pain**	21. Pain magnitude	0.575	0.703
	22. Pain interfere with work	0.611	0.653
	52. Pain interfere with enjoyment	0.601	0.652
**Emotional wellbeing**	24. Nervous person	**0.372**	0.531
	25. Down in dumps	0.561	0.584
	26. Peaceful	0.563	**0.368**
	28. Blue/Sad	0.594	0.592
	30. Happy	0.536	0.431
**Energy**	23. Pep/life	0.714	**0.204**
	27. Energy	0.717	**0.243**
	29. Worn out	0.624	0.546
	31. Tired	0.620	0.602
	32. Rested on walking in the morning	0.519	**0.280**
**Health perceptions**	1. EVGFP rating	0.638	0.451
	34. Sick easier	0.417	**0.269**
	35. As healthy	0.464	0.574
	36. Health to get worse	0.450	**0.233**
	37. Health excellent	0.590	0.658
**Cognitive function**	42. Concentration and thinking	0.592	0.710
	43. Sustained attention	0.576	0.700
	44. Memory	0.468	0.708
	45. Others note troubles with memory/concentration	0.436	0.564
**Health distress**	38. Discouraged	0.729	0.508
	39. Frustrated	0.712	0.544
	40. Worried for life	0.624	0.543
	41. Weighed down	0.694	0.563
**Sexual function**	46. Lack if sexual interest	**0.346**	0.684
	47. Erection/Lubrication	**0.299**	0.758
	48. Orgasm	**0.348**	0.724
	49. Satisfy sexual partner	**0.377**	0.657
**Social function**	20. Social extent, physical health	0.735	0.873
	33. Social time	0.757	**0.209**
	51. Social extent, bowel or bladder	0.502	**0.030**
**Overall quality of life**	53. 0-10 NRS rating	0.735	-
	54. TUMMMPO rating	0.685	-

EVGFP, Excellent, Very good, Good, Fair, Poor. HRQOL, health-related quality of life. NRS, Numeric Rating Scale. TUMMMPO, Terrible, Unhappy, Mostly dissatisfied, Mixed – about equally satisfied and dissatisfied, Pleased, Delighted.

Correlations between residuals: 0.524 (items 53 and 54).

Coefficients <0.40 are reported in bold; all the loadings are statistically significant at $p < 0.001$, with the exception of the three items of the social function subscale that are not statistically significant.
