# Supplementary material for: Viability of a MSQOL-54 general health-related quality of life score using bifactor model
Source: Health Qual Life Outcomes. 2021 Sep 25;19:224. doi: 10.1186/s12955-021-01857-y (PMC8467164; doi:10.1186/s12955-021-01857-y)
Supplement: Supplementary file 5 — Additional file 5: Supplementary table 2. Correlations between items of the social function subscale. [file 12955_2021_1857_MOESM5_ESM.pdf]

Additional File 5

Supplementary Table 2. Correlations between items of the social function subscale.

	Item 20	Item 33	Item 51
Item 20	1	0.62	0.24
Item 33	0.74	1	0.22
Item 51	0.40	0.39	1

Lower-triangular part: zero order correlations; upper-triangular part: partial correlations controlling for Qol subscale score; all Pearson's coefficients are statistically significant at $p < 0.001$.
